# List of Reviewers 2021

**DOI:** 10.34763/jmotherandchild.20212504.Reviewers2021

**Published:** 2022-09-02

**Reviewers 2021**

**Recenzenci 2021**

**Table:** FIRST NAME and LAST NAME / IMIĘ i NAZWISKO

1.	Dr n. biol. JADWIGA AMBROSZKIEWICZ
2.	Mgr socjol. MARTYNA BÓJKO
3.	Dr hab. n. med. MAGDALENA CHEŁCHOWSKA
4.	Prof. dr hab. JOANNA CHOROSTOWSKA-WYNIMKO
5.	Dr n. med. biol. ELŻBIETA CIARA
6.	Prof. dr hab. n. med. MARCIN CZECH
7.	Dr hab. n. med., Prof. IPCZD JUSTYNA CZECH-KOWALSKA
8.	Dr n. hum. JOANNA DEC-PIETROWSKA
9.	Dr hab. n. med. DOROTA DUNIN-WĄSOWICZ
10.	Dr n. społ. ANNA DZIELSKA
11.	Dr inż. DANUTA GAJEWSKA
12.	Dr n. med. KRZYSZTOF GAŁCZYŃSKI
13.	Prof. DIEGO GOMEZ
14.	Prof. dr hab. n. med. EWA HELWICH
15.	Prof. dr hab. n. pedag. ZBIGNIEW IZDEBSKI
16.	Prof. HELENA JERIČEK KLANŠČEK
17.	Dr hab. n. med., Prof. IGiChP ALEKSANDRA JEZELA-STANEK
18.	Prof. dr hab. n. med. MACIEJ JÓŹWIK
19.	Dr n. o zdrowiu DOROTA KLESZCZEWSKA
20.	Dr hab. n. społ. GRAŻYNA KMITA
21.	Mgr MAGDALENA KORZYCKA
22.	Prof. ANNA KOWALEWSKA
23.	Mgr ALICJA KOZAKIEWICZ
24.	Dr MARTA KUCHARSKA-HAUK
25.	Dr hab. n. med. MARIUSZ KUŹMICKI
26.	Prof. dr hab. n. med. RYSZARD LAUTERBACH
27.	Prof. MICHELA LENZI
28.	Dr hab. KATARZYNA LUBIEWSKA
29.	Dr MARTA MALINOWSKA-CIEŚLIK
30.	Dr n. hum. AGNIESZKA MAŁKOWSKA- SZKUTNIK
31.	Dr hab. n. o zdrowiu, prof. IMiD JOANNA MAZUR
32.	Dr EWA MIERZEJEWSKA
33.	Prof. dr SUJATA MISRA
34.	Dr n. o kult. fiz. HANNA NAŁĘCZ
35.	Dr hab. n. med., Prof. IMiD ANNA OBLACIŃSKA
36.	Dr n. hum. KATARZYNA OKULICZ ‑KOZARYN
37.	Dr hab. n. med., prof. IBM EDYTA PARADOWSKA
38.	Prof. KINGA POLAŃSKA
39.	Mgr JOANNA PRUBAN
40.	Dr PENDYALA RAJKUMARI
41.	Prof. dr hab. med. DOROTA SANDS
42.	Prof. TERESA DOS SANTOS
43.	Dr KAROLINA STĘPIEŃ
44.	Prof. BIRUTE STRUKCINSKIENE
45.	Dr hab. n. o zdr. KATARZYNA SZAMOTULSKA
46.	Dr hab. n. med. KRZYSZTOF SZCZAŁUBA
47.	Dr n. med. WOJCIECH WALAS
48.	Prof. dr hab. n. med. i n. o zdrowiu HALINA WEKER
49.	Dr hab. n. biol. ALEKSANDRA WESOŁOWSKA
50.	Dr hab. n. med., Prof. CMKP MARIA WILIŃSKA
51.	Dr n. med. JULIA ZARĘBA-SZCZUDLIK
52.	Dr n. med .ALEKSANDRA ZAREMBA
53.	Dr n. hum. DOROTA ZAWADZKA

Editorial Board of the Journal of Mother and Child / Medycyna Wieku Rozwojowego would like to thank all the Reviewers for their time and commitment to the evaluation of the works.

Redakcja Journal of Mother and Child / Medycyna Wieku Rozwojowego składa serdeczne podziękowania wszystkim Recenzentom za poświęcony czas i zaangażowanie w oceny prac.

